# Clinical Features and Risk Factors of Uveitis in Korean Children with Juvenile Idiopathic Arthritis: A Retrospective Cohort Study

**DOI:** 10.3390/jcm12103438

**Published:** 2023-05-12

**Authors:** Jinsoo Kim, Min Seon Park, Soonil Kwon, Kwang Nam Kim, Han Wool Kim, Bum-Joo Cho

**Affiliations:** 1 Department of Ophthalmology, Hallym University Sacred Heart Hospital, Hallym University College of Medicine, Anyang 14068, Republic of Korea; 2 Department of Pediatrics, Myongji Hospital, Hanyang University College of Medicine, Goyang 10475, Republic of Korea; 3 Department of Pediatrics, Hallym University Sacred Heart Hospital, Hallym University College of Medicine, Anyang 14068, Republic of Korea

**Keywords:** juvenile idiopathic arthritis, uveitis, risk factor, knee, joint

## Abstract

This study aimed to investigate the clinical features and risk factors of uveitis in Korean children with juvenile idiopathic arthritis (JIA). The medical records of JIA patients diagnosed between 2006 and 2019 and followed up for ≥1 year were retrospectively reviewed, and various factors including laboratory findings were analyzed for the risk of developing uveitis. JIA-associated uveitis (JIA-U) developed in 30 (9.8%) of 306 JIA patients. The mean age at the first uveitis development was 12.4 ± 5.7 years, which was 5.6 ± 3.7 years after the JIA diagnosis. The common JIA subtypes in the uveitis group were oligoarthritis-persistent (33.3%) and enthesitis-related arthritis (30.0%). The uveitis group had more baseline knee joint involvement (76.7% vs. 51.4%), which increased the risk of JIA-U during follow-up (*p* = 0.008). Patients with the oligoarthritis-persistent subtype developed JIA-U more frequently than those without it (20.0% vs. 7.8%; *p* = 0.016). The final visual acuity of JIA-U was tolerable (0.041 ± 0.103 logMAR). In Korean children with JIA, JIA-U may be associated with the oligoarthritis-persistent subtype and knee joint involvement.

## 1. Introduction

Juvenile idiopathic arthritis (JIA) is a heterogeneous group of chronic arthritic conditions that affect children before the age of 16 and persist for at least 6 weeks during childhood [1]. Among children with JIA, uveitis is the most common extra-articular manifestation, affecting 6.1% to 22.1% of patients [2,3,4]. JIA-associated uveitis (JIA-U) is generally insidious and chronic, and thus, it often remains asymptomatic until ocular structural damage occurs, which can lead to sight-threatening complications [3,5]. Nevertheless, it can be also acute and symptomatic in 10–20% of cases, and this type is associated with the enthesitis-related arthritis (ERA) subtype of JIA and older age at uveitis onset [3,6].

Several risk factors have been identified for the development of uveitis in JIA, including earlier onset of JIA, oligoarticular JIA subtypes, female sex, and positivity for antinuclear antibodies (ANA) [3,4,7,8,9]. Conversely, late onset of JIA, rheumatoid factor (RF)-positive polyarthritis, systemic arthritis, and positivity for anticyclic citrullinated peptide (CCP) antibodies are suggested to be protective factors against JIA-U [2,4,8]. Although there are ethnic and geographical differences in the incidence of JIA-U [10,11], few studies have investigated the clinical features and risk factors for JIA-U in Asian or Korean children [2]. Additionally, the relationship between the pattern of joint involvement and the development of uveitis in JIA-U has not been extensively studied. Identifying patients at higher risk of JIA-U is essential for appropriate screening, early diagnosis, and timely treatment of uveitis [8].

Therefore, the purpose of this study was to investigate the clinical features and risk factors of JIA-U among Korean children. In this study, we comprehensively reviewed the medical records of both pediatric rheumatology and ophthalmology departments, including the clinical manifestation and laboratory findings of JIA, for patients recruited at a single tertiary referral center over a 13-year period. To the best of our knowledge, this is one of the largest studies on the risk factors for JIA-U among Asian children and the first analysis of the relationship between the profile of joints involved in JIA and the development of JIA-U.

## 2. Materials and Methods

### 2.1. Study Subjects

This retrospective study included all consecutive Korean children diagnosed with JIA between January 2006 and March 2019 at Hallym University Sacred Heart Hospital, a tertiary referral center. The medical records of subjects from both the pediatric rheumatology and ophthalmology departments were carefully reviewed. Only those who had been followed-up for a minimum of one year in pediatric rheumatology and six months in ophthalmology were included. All eligible patients were assigned to one of two groups based on the occurrence of uveitis at diagnosis or during the follow-up period of JIA: the uveitis group vs. the non-uveitis group. Several clinical factors, including laboratory findings, were examined for the risk of developing uveitis. This study was approved by the Institutional Review Board (IRB No. Hallym 2019-09-022) of Hallym University Sacred Heart Hospital and was performed in accordance with the principles of the Declaration of Helsinki. Patient consent was waived because of the retrospective nature of the study and the minimal level of risk arising from the study.

### 2.2. Main Clinical Outcomes

The demographic variables of the patients and the clinical characteristics of JIA, including subtype and involved joints, were carefully investigated. The JIA subtype was determined according to the International League of Associations for Rheumatology classification, which consists of seven categories: systemic arthritis, oligoarthritis (persistent or extended), polyarthritis RF-positive, polyarthritis RF-negative, ERA, psoriatic arthritis, and undifferentiated arthritis, which did not meet any or met more than one criterion [12]. In each patient, joint involvement in JIA was evaluated for hand, wrist, elbow, shoulder, foot, ankle, knee, hip, neck, back, and temporomandibular joints for all involved joints with swelling or tenderness, both upon diagnosis and during follow-up.

Laboratory findings at baseline were also reviewed for ANA, RF, anti-CCP antibody, human leukocyte antigen (HLA) B-27, erythrocyte sedimentation rate (ESR), and C-reactive protein (CRP). The patients were considered ANA-positive if they had at least two positive results on indirect immunofluorescence assays performed over 3 months apart [9,13]. HEp-2 cells were the substrate used for ANA determination. RF and anti-CCP Ab were all tested by enzyme-linked immunosorbent assay. The patients were considered RF-positive if they had at least two positive results (≥20 IU/mL) over 3 months apart. The patients were considered anti-CCP Ab-positive if there was at least one positive test (≥20 AU/mL). Treatment regimens, including the use of immunomodulatory agents and biological agents during follow-up, were also investigated.

All JIA patients at the department of pediatric rheumatology were immediately checked for uveitis by an ophthalmologist at the same hospital. All patients underwent ophthalmologic examination, including measurement of visual acuity (VA), intraocular pressure, manual refraction, slit-lamp biomicroscopy, and dilated fundus examination. Fluorescein angiography or optical coherence tomography was not routinely performed. The clinical characteristics of JIA-U, including age at diagnosis, laterality at initial diagnosis and at recurrence, location, treatment regimens, ocular complications, and ophthalmic surgeries, were analyzed. The classification criteria and grading system suggested by the standardization of the uveitis nomenclature working group were used to diagnose and evaluate uveitis [5,14]. Uveitis recurrence was defined as a repeated episode of active inflammation separated by a period of at least 3 months of inactivity. Inactive anterior uveitis was defined as the absence of anterior chamber cells [14].

### 2.3. Statistical Analysis

Continuous variables are expressed as means ± standard deviation; categorical variables are expressed as numbers and proportions among the whole. VA was estimated as the logarithm of the minimum angle of resolution (logMAR). The Wilcoxon signed-rank test was used to compare the initial and final VA. The chi-square test was used to compare categorical variables. The Mann–Whitney test was performed to analyze the between-group difference in the grade of inflammatory cells in the anterior chamber. If the expected values were less than 5, Fisher’s exact test was used to analyze the association between categorical variables. An independent t-test was used to compare other continuous variables. The Kruskal–Wallis test was used to compare age at JIA diagnosis between JIA subtypes, and pairwise comparisons were conducted by the Dunn–Bonferroni post hoc method. Kaplan–Meier analysis was used to analyze the development of uveitis during the follow-up period by the joint involved at the onset of JIA. The *p*-value was calculated by comparing the two survival curves using the log-rank test. Statistical significance was set at *p* < 0.05. All analyses were performed using the IBM Statistical Package for the Social Sciences software version 26 (IBM Corporation, Armonk, NY, USA).

## 3. Results

### 3.1. Baseline Characteristics of JIA Patients at Diagnosis

**Figure 1:**
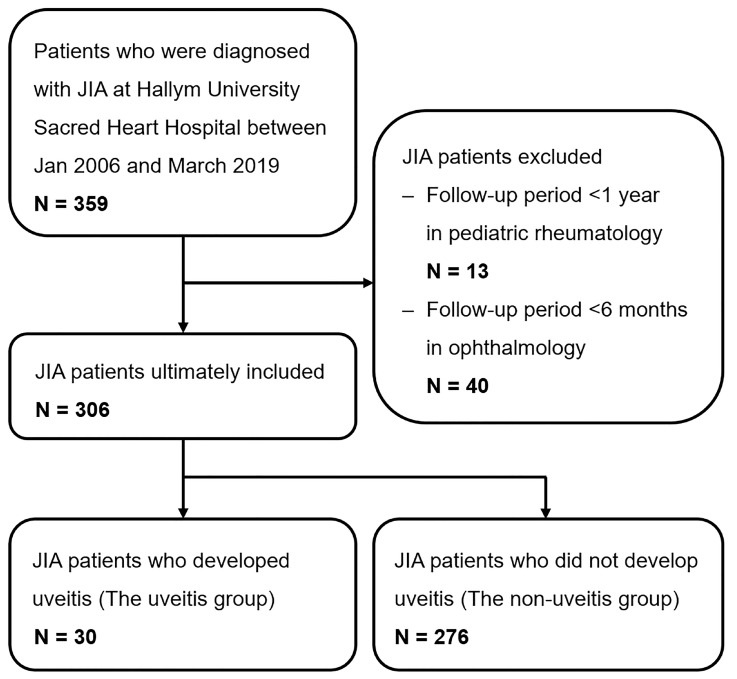
Participation flowchart for the enrollment of patients with juvenile idiopathic arthritis. JIA: juvenile idiopathic arthritis.

**Figure 2:**
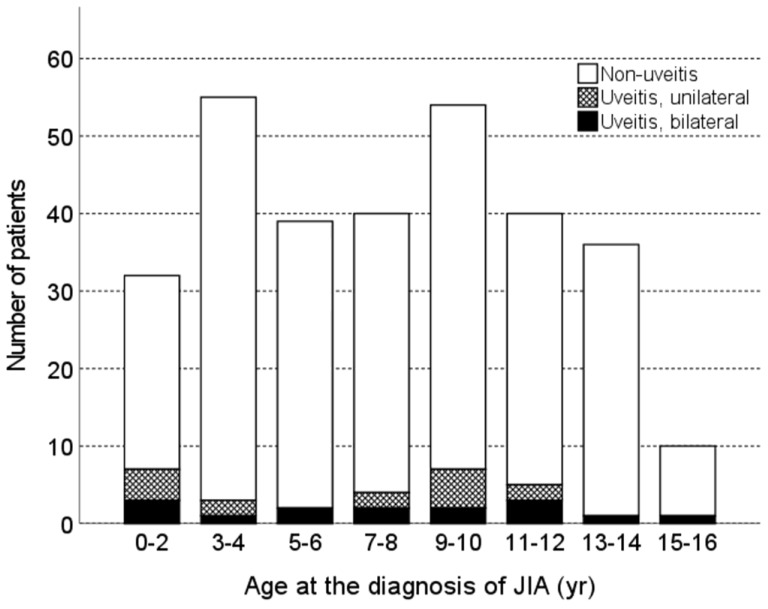
Age distribution of juvenile idiopathic arthritis patients with or without uveitis. JIA: juvenile idiopathic arthritis.

A total of 306 Korean children with JIA were enrolled in this study, and the enrollment process is illustrated in Figure 1. The mean age at diagnosis of JIA was 7.7 ± 4.0 years, with a median age of 7.6 years. JIA-U developed in 30 (9.8%) patients during the follow-up period. The baseline characteristics of the patients in the uveitis and non-uveitis groups are presented in Table 1. The follow-up period was significantly longer in the uveitis group than in the non-uveitis group (10.1 ± 4.7 vs. 8.1 ± 4.6; *p* = 0.019). The proportion of male patients was 43.3% in the uveitis group and 48.2% in the non-uveitis group, but the difference was not statistically significant (*p* = 0.613). There was no significant difference in age at JIA diagnosis between the groups (*p* = 0.626). However, age at JIA diagnosis showed a significant difference between JIA subtypes (*p* < 0.001), and pairwise comparison between the oligoarthritis-persistent subtype and ERA showed statistical significance (5.5 ± 4.0 vs. 9.9 ± 2.6; adjusted *p* < 0.001). Figure 2 shows the age distribution of JIA patients in the uveitis and non-uveitis groups.

The most common JIA subtype in the uveitis group was oligoarthritis-persistent (33.3%), followed by ERA (30.0%). Patients with the oligoarthritis-persistent subtype were more likely to belong to the uveitis group than those with other subtypes (20.0% vs. 7.8%; *p* = 0.016). In contrast, patients with systemic arthritis were significantly less likely to belong to the uveitis group than those without (3.6% vs. 12.2%; *p* = 0.024). JIA-U developed in 16.7% (3 of 18) of oligoarthritis-extended patients and 13.6% (9 of 66) of ERA patients. JIA-U was not observed in any patient with RF-positive polyarthritis or psoriatic arthritis.

In terms of laboratory findings, the rate of presence of ANA was higher in the uveitis group than in the non-uveitis group, although the statistical significance was only marginal (*p* = 0.057). The presence of RF was lower in the uveitis group, but there was no significant difference between the two groups (*p* = 0.096). Of note, JIA-U did not develop in 37 patients who were positive for anti-CCP antibody. ESR was significantly higher in the uveitis group compared with the non-uveitis group (*p* = 0.036).

### 3.2. Clinical Features and Treatment Outcomes of JIA-U

The clinical features of 30 patients with JIA-U are presented in Table 2. The mean age at diagnosis of JIA-U was 12.4 ± 5.7 years, and the median age was 12.9 years. In 3 (10.0%) patients, uveitis existed before or at the time of JIA diagnosis, and among the others, the mean period from the diagnosis of JIA to the diagnosis of JIA-U was 5.6 ± 3.7 years.

JIA-U was recurrent in 15 (50.0%) patients, including all the three patients with the oligoarthritis-extended subtype and none of the three patients with the systemic arthritis subtype.

The mean grade of inflammatory cells in the anterior chamber was 1.7 ± 1.1 at the initial diagnosis of JIA-U, which was not significantly different between children under 8 years old and those over that age (*p* = 0.595). The mean initial VA at the first uveitis occurrence was 0.149 ± 0.355 logMAR, and the mean final VA of these eyes was 0.041 ± 0.103 logMAR. The final VA was significantly better than the initial VA (*p* = 0.036).

Treatment regimen and ocular complications of JIA-U are presented in Table 3.

All patients with JIA-U received topical steroids (prednisolone 1%, administered every 2 h with tapering according to degree of inflammation), while 10 patients also received oral steroids (prednisolone, initially 1 mg/kg/day with tapering to 0.15 mg/kg/day within 4–5 weeks). Methotrexate (93.3%) was the most frequently used immunomodulatory drug administered orally at a dosage of 10–15 mg/m^2^/week, followed by hydroxychloroquine (20.0%) used orally and adjunctively with methotrexate at a dosage of 1 mg/kg/day. Among anti-tumor necrosis factor (TNF) agents, etanercept was prescribed more often than adalimumab (15 vs. 6).

Posterior synechiae (7 patients, 10 eyes) were the most common complication, followed by band keratopathy (5 patients, 8 eyes), cataracts (4 patients, 7 eyes), and glaucoma (2 patients, 3 eyes). Scleral involvement was not found. Ophthalmic surgery was performed on 7 eyes (5 patients), of which 4 eyes (3 patients) underwent cataract surgery and 3 eyes (2 patients) underwent Ahmed valve implantation.

### 3.3. Baseline Joint Involvement of JIA Patients According to the Uveitis Development

The joint involvement profiles of the uveitis and non-uveitis groups upon JIA diagnosis are shown in Table 4. Knee joints were the most commonly involved in both groups. At baseline, knee joint involvement was seen in 165 (53.9%) out of the 306 patients, and shoulder joint involvement was present in 34 (11.1%) patients. The uveitis group had a significantly higher rate of knee joint involvement than the non-uveitis group (*p* = 0.008), while no shoulder joint involvement was observed in the uveitis group, in contrast to the non-uveitis group (*p* = 0.041).

Of 165 patients with knee joint involvement at baseline, 23 (13.9%) developed JIA-U during the follow-up period, while only 7 (5.0%) out of 141 patients without knee joint involvement developed uveitis (*p* = 0.008). Moreover, none of the 34 patients with shoulder joint involvement developed JIA-U, while 30 (11.0%) out of 272 patients without shoulder involvement developed JIA-U (*p* = 0.034). In the oligoarthritis-persistent subtype, there was no difference in knee joint involvement and shoulder joint involvement between the uveitis group and the non-uveitis group (*p* = 0.702 and >0.999, respectively).

**Figure 3:**
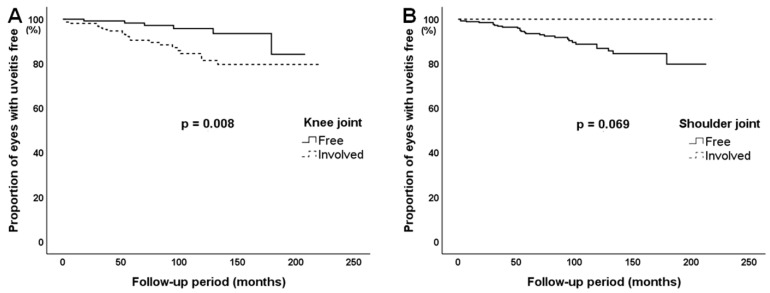
Survival curves for uveitis development in patients with juvenile idiopathic arthritis according to the involvement of (**A**) knee and (**B**) shoulder joints at baseline.

The Kaplan–Meier survival curves for knee and shoulder joints are depicted in Figure 3. During the follow-up period, uveitis occurred more frequently in JIA patients with knee joint involvement than in those without knee joint involvement (*p* = 0.008). However, there was no significant difference in the occurrence of uveitis in patients with shoulder joint involvement (*p* = 0.069).

## 4. Discussion

The present study investigated the clinical features and risk factors for the development of uveitis in 306 Korean children with JIA who were recruited over a period of 13 years. The study found that the oligoarthritis-persistent subtype and knee joint involvement at JIA diagnosis were associated with the development of uveitis. However, JIA patients with shoulder joint involvement at diagnosis did not develop JIA-U during the follow-up period.

In this study, the rate of uveitis development among JIA patients was 9.8%, which was slightly higher than that in Japan (6.1%) but lower than that in the United States and the Nordic regions (11.6% and 22%, respectively) [2,4]. This is consistent with previous studies reporting lower JIA-U prevalence in Asians than in Caucasians [10,11]. On the other hand, the age at JIA diagnosis was higher than that in other studies; for example, the mean age was 6.4 years in the United States [4], the median age was 5.7 years in the Nordic region [3], and the median age was 5.5 years in Japan [2]. Although early onset of JIA has been reported as a risk factor for the development of uveitis [2,4], there was no difference in age at diagnosis of JIA between the uveitis group and the non-uveitis group. These discrepancies with other studies might be explained by the large proportion of patients with ERA, which is more frequent in older children [15].

In terms of JIA subtypes, the oligoarthritis-persistent subtype was more frequent in the uveitis group than the non-uveitis group (33.3% vs. 14.5%) in our study, which is consistent with previous studies [2,4]. The oligoarthritis subtype of JIA has long been recognized as a significant risk factor for JIA-U, accounting for 76.5% of JIA-U patients in previous studies [16,17]. Additionally, the ERA subtype accounted for a substantial proportion of the uveitis group in this study, as seen in previous Asian studies [18]. So far, the association between JIA-U and the oligoarthritis or ERA subtype of JIA remains incompletely explained. Nonetheless, it is advisable to schedule frequent ophthalmologic examinations for oligoarthritis patients [19,20].

Of note, our study demonstrated a significantly higher rate of knee joint involvement and a significantly lower rate of shoulder joint involvement in the uveitis group compared with the non-uveitis group, although the knee joint was the most commonly involved joint in both groups, as shown in a previous study [21]. In this study, the involvement of the knee joint at diagnosis of JIA was found to increase the risk of JIA-U during the follow-up period. It is challenging to explain why the knee joint can act as a risk factor for JIA-U, and there may be a potential immunological association between the eye and the knee. Considering the significant difference in knee joint involvement and shoulder joint involvement between the group with uveitis and without uveitis among JIA patients, and the absence of differences in the oligoarthritis-persistent subtype, it is difficult to determine knee joint involvement as a direct risk factor for JIA-U. In a previous study, patients with oligoarthritis and RF-negative polyarthritis demonstrated greater involvement of the knee joint and less involvement of the shoulder joint in the ANA-positive group compared with the ANA-negative group [22]. Similarly, the patients who were positive for ANA demonstrated a higher occurrence of uveitis and displayed less frequent involvement of the shoulder joints [23]. Therefore, the increased rate of knee joint involvement in the uveitis group could be attributed to the prevalence of the ANA-positive oligoarthritis subtype among these patients. Additional research is needed to uncover the underlying pathogenesis.

Thus far, ANA positivity has been reported to be higher in the uveitis group in several studies, sometimes showing an odds ratio of 1.88 [8,17,20]. However, there was no statistical significance in this study, which might be due to the insufficient number of subjects, as in other studies [7]. ANA positivity increased the risk of uveitis development (30% vs. 3%) regardless of JIA subtype [23], and the underlying cause is yet to be elucidated. In contrast, the anti-CCP antibody was negative in JIA-U patients in this study, as in previous studies [2,4,8]. Anti-CCP antibody positivity is more common in RF-positive polyarthritis, which has an aggressive and erosive disease course but a lower occurrence rate of uveitis [24,25]. Consistent with previous studies that reported RF-positive polyarthritis was protective against JIA-U [4,20], uveitis did not occur in RF-positive polyarthritis in our study. Therefore, the anti-CCP negativity of the uveitis group might reflect the absence of RF-positive polyarthritis in that group. HLA B-27 is associated with ERA, which mainly presents as acute uveitis [5,26]; its positivity was slightly higher in the uveitis group, although it was not statistically significant due to an insufficient number of subjects. As previously reported, the elevated ESR at the diagnosis of JIA, which could reflect a high level of inflammation and a hyperactive immune system at baseline, was associated with the development of JIA-U [27,28].

The most commonly used treatment in the uveitis group was methotrexate, except for topical steroid eye drops, which were administered to all patients. In our study, etanercept was favored over adalimumab and infliximab, despite its lower efficacy compared with adalimumab and infliximab in treating JIA-U [29,30]. This could be attributed to the fact that the study included a substantial number of patients from the period prior to the availability of adalimumab. Additionally, the treatment outcome of etanercept alone was found to be quite favorable, as previously reported [31].

The final visual outcome in this study was 0.041 logMAR, which was comparable to the visual outcome reported in previous studies (0.11 logMAR) after a 2-year follow-up of uveitis [32]. Our study demonstrated a lower rate of ocular complications, specifically cataracts (15.9% vs. 23.7%) and glaucoma (6.8% vs. 22.2%), compared with recent reports [3]. One possible explanation for the reduced complication rate could be the inclusion of more recent patients and a shorter follow-up period in our study. Additionally, our study involved immediate consultation between a pediatric rheumatologist and the ophthalmology department upon the initial diagnosis of JIA, suggesting that effective collaboration between these specialties may contribute to improved visual outcomes and a decrease in sight-threatening complications. Furthermore, the higher proportion of patients with ERA exhibiting uveitis and experiencing fewer ocular complications might be a third contributing factor [5].

Our study had several limitations due to its retrospective design. Since the enrollment period spanned up to 10 years, the treatment regimen varied, with etanercept being the most commonly used anti-TNF agent. As a result, the results may not accurately reflect the recent treatment outcomes using adalimumab. Furthermore, being a single-center study, the sample size of the uveitis group was not sufficiently large enough to yield statistically significant differences in some examinations. In addition, all the subjects in our study were Asians, so the findings may not directly apply to other ethnicities.

## 5. Conclusions

This study found that the development of JIA-U is positively associated with the oligoarthritis-persistent subtype and inversely associated with systemic arthritis in Korean children with JIA. Baseline knee joint involvement at the time of JIA diagnosis was also associated with the development of uveitis, and further research is required to explore this association. The epidemiology and clinical features identified in this study could be helpful in the screening, treatment, and follow-up of children with JIA.

## Figures and Tables

**Table 1:** Baseline characteristics of Korean children diagnosed with JIA.

Characteristics	All Patients (*n* = 306)	Uveitis Group (*n* = 30)	Non-Uveitis Group (*n* = 276)	*p*-Value
Male, *n* (%)	146 (47.7%)	13 (43.3%)	133 (48.2%)	0.613 ^a^
Age at the JIA diagnosis (yrs)	7.7 ± 4.0	7.4 ± 4.3	7.7 ± 4.0	0.626 ^b^
Follow-up period (yrs)	8.3 ± 4.6	10.1± 4.7	8.1± 4.6	0.019 ^b^
**JIA subtype, *n* (%)**				
Systemic arthritis	84 (27.5%)	3 (10.0%)	81 (29.3%)	0.024 ^a^
Oligoarthritis-persistent	50 (16.3%)	10 (33.3%)	40 (14.5%)	0.016 ^c^
Oligoarthritis-extended	18 (5.9%)	3 (10.0%)	15 (5.4%)	0.401 ^c^
RF-negative polyarthritis	45 (14.7%)	4 (13.3%)	41 (14.9%)	>0.999 ^c^
RF-positive polyarthritis	22 (7.2%)	0 (0%)	22 (8.0%)	0.146 ^c^
Psoriatic arthritis	2 (0.7%)	0 (0%)	2 (0.7%)	>0.999 ^c^
Enthesitis-related arthritis	66 (21.6%)	9 (30.0%)	57 (20.7%)	0.237 ^a^
Undifferentiated arthritis	19 (6.2%)	1 (3.3%)	18 (6.5%)	0.706 ^c^
**Laboratory findings**				
ANA positivity, tested *n* = 306	96 (31.4%)	14 (46.7%)	82 (29.7%)	0.057 ^a^
RF positivity, tested *n* = 306	44 (14.4%)	1 (3.3%)	43 (15.6%)	0.096 ^c^
Anti-CCP positivity, tested *n* = 294	37 (12.6%)	0 (0%)	37 (14.0%)	0.020 ^c^
HLA-B27 positivity, tested *n* = 303	95 (31.4%)	11 (36.7%)	84 (30.8%)	0.509 ^a^
ESR (mm/H), tested *n* = 262	42.0 ± 30.4	54.4 ± 31.1	40.7 ± 30.2	0.036 ^b^
CRP (mg/L), tested *n* = 260	30.4 ± 42.2	45.6 ± 52.3	28.8 ± 40.8	0.063 ^b^

JIA: juvenile idiopathic arthritis; RF: rheumatoid factor; ANA: antinuclear antibody, anti-CCP anticyclic citrullinated peptide antibody; HLA: human leukocyte antigen; ESR: erythrocyte sedimentation rate; CRP: C-reactive protein. ^a^ Chi-square test; ^b^ independent t-test; ^c^ Fisher’s exact test.

**Table 2:** Clinical features of patients with JIA-associated uveitis.

Variables	
Age at the first development of uveitis (yrs)	12.4 ± 5.7
Follow-up period after the first development of uveitis (yrs)	5.10 ± 4.21
Period from the JIA diagnosis to the uveitis development (yrs)	5.6 ± 3.7
Prior to diagnosis of JIA	3 (10.0%)
Initial visual acuity (logMAR), *n* = 36 eyes	0.149 ± 0.355
Final visual acuity (logMAR), *n* = 36 eyes	0.041 ± 0.103
**Clinical course of uveitis**	
Acute	4 (13.3%)
Recurrent	15 (50.0%)
Chronic	11 (36.7%)
**Location of uveitis**	
Anterior uveitis	27 (90.0%)
Intermediate uveitis	0
Posterior uveitis	0
Pan uveitis	3 (10.0%)

JIA: juvenile idiopathic arthritis; logMAR: logarithm of the minimum angle of resolution.

**Table 3:** Treatment regimen and ocular complications of patients with JIA-associated uveitis.

Variables	
**Treatment regimen ever used**	
Topical steroid	30
Subconjunctival dexamethasone injection	2
Oral steroid	10
DMARDs	
Methotrexate	28
Hydroxycholoroquine	6
Sulfasalazine	2
Azathioprine	1
Anti-TNF agents	
Etanercept	15
Adalimumab	6
**Ocular complications**	
Any of the followings	8 patients, 10 eyes (22.7%)
Posterior synechiae	7 patients, 10 eyes (22.7%)
Cataract	4 patients, 7 eyes (15.9%)
Glaucoma	2 patients, 3 eyes (6.8%)
Band keratopathy	5 patients, 8 eyes (18.2%)
**Surgical intervention**	
Cataract surgery	3 patients, 4 eyes (9.1%)
Glaucoma surgery	2 patients, 3 eyes (6.8%)

DMARD: disease modifying antirheumatic drug; TNF: tumor necrosis factor.

**Table 4:** Baseline joint involvement at diagnosis of JIA patients according to uveitis development.

Joints	All Patients (*n* = 306)	Uveitis Group (*n* = 30)	Non-Uveitis Group (*n* = 276)	*p*-Value
Hand	85 (27.8%)	6 (20.0%)	79 (28.6%)	0.317 ^a^
Wrist	74 (24.2%)	3 (10.0%)	71 (25.7%)	0.056 ^a^
Elbow	51 (16.7%)	6 (20.0%)	45 (16.3%)	0.606 ^a^
Shoulder	34 (11.1%)	0 (0%)	34 (12.3%)	0.041 ^a^
Foot	73 (23.9%)	7 (23.3%)	66 (23.9%)	0.944 ^a^
Ankle	133 (43.5%)	13 (43.3%)	120 (43.5%)	0.988 ^a^
Knee	165 (53.9%)	23 (76.7%)	142 (51.4%)	0.008 ^a^
Hip	44 (14.4%)	7 (23.3%)	37 (13.4%)	0.167 ^b^
TMJ	9 (2.9%)	1 (3.3%)	8 (2.9%)	>0.999 ^b^
Neck	4 (1.3%)	0 (0%)	4 (1.4%)	>0.999 ^b^
Back	4 (1.3%)	0 (0%)	4 (1.3%)	>0.999 ^b^

JIA: juvenile idiopathic arthritis; TMJ: temporomandibular joint. ^a^ Chi-square test; ^b^ Fisher’s exact test.

## Data Availability

The data presented in this study are available on reasonable request from the corresponding author. The data are not publicly available due to a lack of patients’ consent to public data sharing.
